# Frog Skin Innate Immune Defences: Sensing and Surviving Pathogens

**DOI:** 10.3389/fimmu.2018.03128

**Published:** 2019-01-14

**Authors:** Joseph F. A. Varga, Maxwell P. Bui-Marinos, Barbara A. Katzenback

**Affiliations:** Department of Biology, University of Waterloo, Waterloo, ON, Canada

**Keywords:** amphibian, anuran, epithelial cells, mucosal tissue, antimicrobial peptides (AMPs), pattern recognition receptors (PRRs), skin microbiome, skin immunology

## Abstract

Amphibian skin is a mucosal surface in direct and continuous contact with a microbially diverse and laden aquatic and/or terrestrial environment. As such, frog skin is an important innate immune organ and first line of defence against pathogens in the environment. Critical to the innate immune functions of frog skin are the maintenance of physical, chemical, cellular, and microbiological barriers and the complex network of interactions that occur across all the barriers. Despite the global decline in amphibian populations, largely as a result of emerging infectious diseases, we understand little regarding the cellular and molecular mechanisms that underlie the innate immune function of amphibian skin and defence against pathogens. In this review, we discuss the structure, cell composition and cellular junctions that contribute to the skin physical barrier, the antimicrobial peptide arsenal that, in part, comprises the chemical barrier, the pattern recognition receptors involved in recognizing pathogens and initiating innate immune responses in the skin, and the contribution of commensal microbes on the skin to pathogen defence. We briefly discuss the influence of environmental abiotic factors (natural and anthropogenic) and pathogens on the immunocompetency of frog skin defences. Although some aspects of frog innate immunity, such as antimicrobial peptides are well-studied; other components and how they contribute to the skin innate immune barrier, are lacking. Elucidating the complex network of interactions occurring at the interface of the frog's external and internal environments will yield insight into the crucial role amphibian skin plays in host defence and the environmental factors leading to compromised barrier integrity, disease, and host mortality.

## Introduction

Nearly 8,000 amphibian species have been discovered to date (88% belonging to order Anura–frogs and toads) and approximately 150 new species are discovered each year (1). Collectively, frogs have evolved unique skin adaptations to live in aquatic and terrestrial environments (2, 3), while exhibiting common elements in their skin composition and structure (4–6). Skin is an integral interface between an organism's internal and external environment and undergoes routine exposure to a myriad of environmental factors, including pathogen challenge. Frog skin is no exception; it acts as a critical immune organ constituting a complex network of physical, chemical, immunological, and microbiological barriers to pathogen insult. Striking commonalities exist between frog, fish, and mammalian skin and exemplify the importance of endeavours in comparative vertebrate skin biology to address numerous research areas (7, 8). As a consequence of their reliance on terrestrial or aquatic habitats, or a combination thereof, amphibian skin is a sophisticated mucosal organ with specialized adaptations required to perform various critical physiological functions (e.g., ion transport, respiration, water uptake, etc.), while still maintaining a selective barrier to the external environment (2, 3, 9). Other than the presence of a sophisticated glandular system, a miraculous feature of amphibian skin that sets frogs apart from other vertebrates is their ability to rapidly heal deep wounds which protrude through the dermal layers without scar formation, including complete regeneration of any glands affected by the injury (8). Despite extensive studies showing that amphibian skin is vital to survival, and apart from antimicrobial peptides (AMPs) (10, 11), relatively little focus has been placed on examining the role of frog skin epithelium to pathogen defence. This focus is paramount since mucosal epithelia are more prone to pathogen attack, such as that seen in mammalian lung and gut epithelium (12–15). With the rise of declining amphibian populations globally (16), wherein emerging infectious diseases such as frog virus-3 (FV3), the type species of the genus *Ranavirus* (family *Iridoviridae*), and the fungal pathogen *Batrachochytrium dendrobatidis (Bd)* (17, 18) are believed to be the proximal cause (19, 20). It is therefore important to understand the interplay between frog skin, pathogens and contributing environmental factors.

## Amphibian Skin—the First Barrier of Defence

Maintenance of amphibian skin integrity is important for overall frog health—both in terms of conducting essential physiological processes and for defence against invading pathogens. Depending on the species, amphibian skin contributes to water uptake, ion transport, respiration, heat transfer, camouflage, and predator deterrence (9). Yet frog skin is particularly vulnerable to cutaneous injury due to the relatively thin and permeable nature of the organ—characteristics necessary to support many of the aforementioned physiological processes. Thus, frog skin is an important first line of defence against harmful agents in the environment that may disrupt skin function and/or cause cutaneous or systemic diseases, leading to interruption of essential physiological functions and ultimately amphibian death. In addition to common innate immune elements, amphibians have evolved specialized features to enhance innate immune defences to protect the vulnerable skin barrier, including a glandular network beneath the skin surface that are capable of producing a plethora of antimicrobial and toxic substances, thus aiding in the defence against pathogens and predators (6). While much remains to be elucidated, the holism between amphibian skin, host physiology and immunity is apparent.

### Skin Layer Organization and Composition

**Figure 1:**
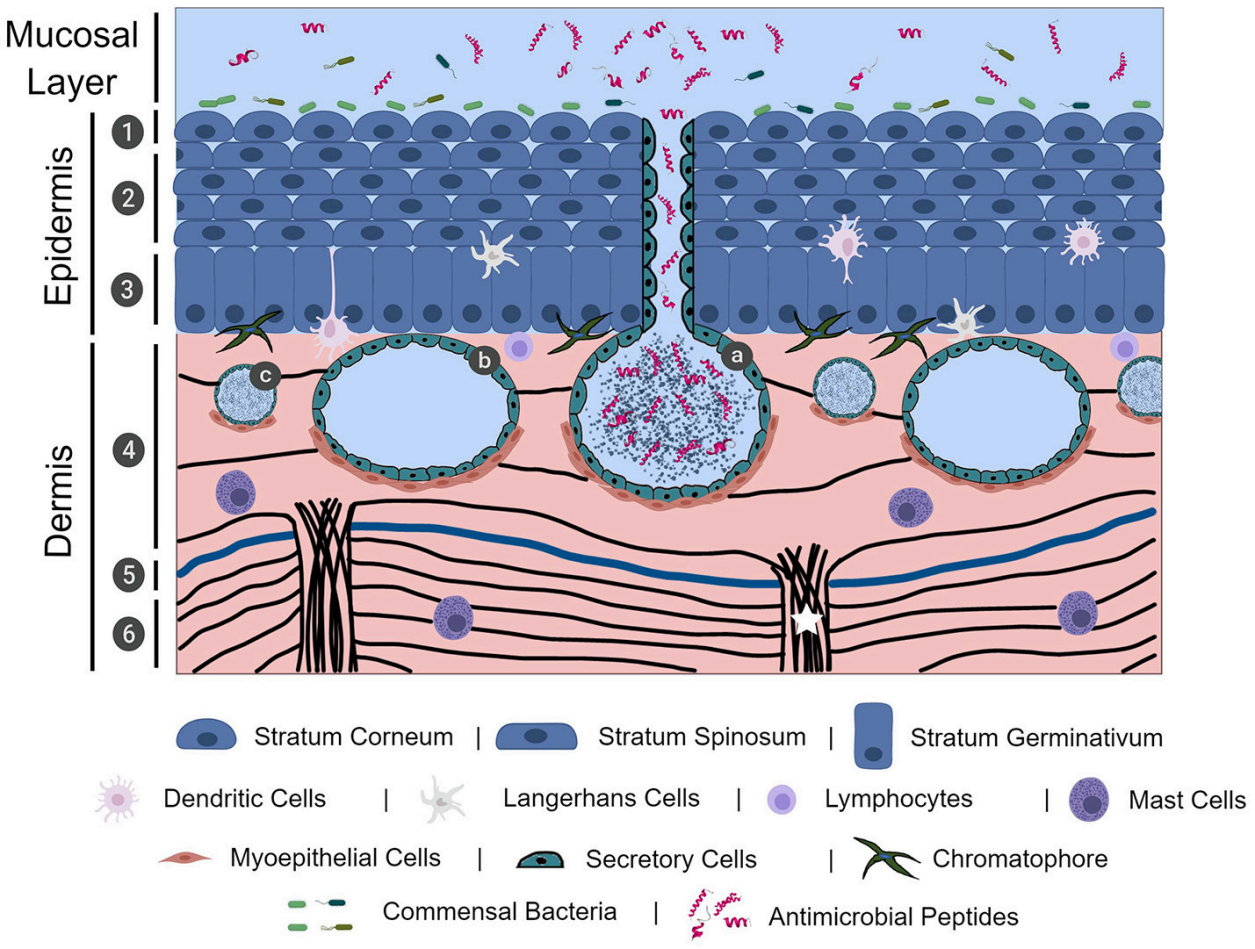
The physical, chemical, cellular, and microbiological innate immune barriers of frog skin. Frog skin, which is mucosal in nature, contains physical, chemical, cellular, and microbiological barriers that work together in defence against pathogen assault. Frog skin is composed of an epidermal and dermal layer, containing resident immune cells throughout the layers. The epidermis is comprised of stratified squamous epithelial cells in three distinct layers: the stratum corneum (1), stratum spinosum (2), and stratum germinativum. The dermis is largely comprised of connective tissue formed by collagenous fibres (black lines) in two layers, the spongious dermis (4) and the compact dermis (6), connected by collagenous columns (white star). In mainly terrestrial frogs, the Eberth-Kastschenko layer (5, thick blue line) separates the spongious dermal layer and compact dermal layer. Glands within the dermal layer include granular glands (a), mucosal glands (b), and small mixed glands (c) that secrete a slew of compounds, including mucus and antimicrobial peptides. Commensal bacteria overlay the frog skin layers, forming the microbiological barrier. The image was, in part, created with the aid of BioRender.

Frog skin is composed of epidermal and dermal layers, with each layer predominantly consisting of epithelial and fibroblastic cells, respectively. While mammalian epidermal strata layers are well-defined due to its thickness, frog epidermis is relatively thin and thus often limited to the stratum corneum (outermost layer), central stratum spinosum, and stratum germinativum (basal layer) (Figure 1) (7). Frog epidermis is composed of stratified squamous epithelium, wherein the stratum corneum is composed of a very thin layer of keratinized cells (Figure 1) (7, 21). Cells in the epidermis of tadpoles are ciliated in most of the frog species studied and cilia regress leading up to metamorphosis. In general, this is characterized by a global loss of ciliated skin cells at Gosner stages 25–30 with the exception of the retention of cilia around the eye and nasal areas (22, 23). To date, there are no studies on the importance of the mucociliary epithelium in adult frogs. We presume the mucociliary function in amphibians is similar to that of other organisms, where the cilia play an important role in sweeping trapped microbes away from mucosal surfaces (24, 25). The stratum spinosum is composed of terminally differentiating cells, acting as an intermediate layer between the stratum corneum and the regenerative stratum germinativum layer (7). The stratum germinativum, which directly connects to the dermis, contains a mixture of cell types including epithelial cells, immune cells (described in the paragraph immediately below) and chromatophores that provide frogs with dynamic pigmentation patterns (26). The dermal layer can be divided into two distinct layers: the upper spongious dermis and lower compact dermis (Figure 1). The spongious dermal layer is composed of loose connective tissue, while the compact dermal layer is formed by a series of interweaving collagenous fibre bundles, with fibronectin situated between breaks in the collagenous layers (Figure 1) (27, 28). Fibroblastic cells, which produce collagenous fibres to form connective tissue, are integral in anchoring the epidermal and dermal layers to the hypodermis particularly through collagenous columns (Figure 1) (27). A unique feature to select, mainly terrestrial, adult amphibian dermis is the separation of spongious and compact dermis by the Eberth-Katschenko (EK) layer (Figure 1) (5). This non-cellular layer is composed entirely of glycosaminoglycans and glycoconjugates, wherein hyaluronan and dermatan sulphate have been reported as key constituents in various species (29, 30). Hyaluronan and other hyaluronan-like molecules in the EK layers are predominantly found on the dorsal side of amphibian skin. Hyaluronan molecules are proposed to reduce water evaporation thereby aiding in the prevention of desiccation, particularly in basking amphibians, since the molecules are highly water retentive (30). In addition to the EK divide, thick collagenous columns extend upwards from the hypodermis to the spongious dermis layer, without impacting the compact dermis integrity, and functions to anchor the layers of skin (27). This anchoring is completed by hemidesmosomes that connect epidermal cytoskeletal filaments to dermal collagenous fibrils (31). The epidermal and dermal layers are essential to the overall integrity of amphibian skin.

Though not largely studied, and thus not often described in studies examining frog integument, resident immune cells responsible for detecting and responding to pathogen exposure have been identified in frog skin (Figure 1). Intertwined amongst the skin epidermal cells of American bullfrogs, (*Rana catesbeiana* syn. *Lithobates catesbeiana)*, Northern leopard frogs, (*Rana pipiens* syn. *Lithobates pipiens*), and African clawed frogs, (*Xenopus laevis*) are dendritic-like or Langerhans-like cells (32–34). Mast cells play an important role in inflammatory and anti-parasitic responses via degranulation of biologically-active compounds, such as histamine, (35) and have also been identified in histological preparations of *R. catesbeiana* skin tissues (29). Less common in the literature are sporadic reports on the presence of macrophages and lymphocytes in healthy skin tissue (7). However, during epithelial wounding or pathogen insult, recruitment of circulating immune cells to the site can occur. Although T cells do not appear to be resident in the skin tissues of frog species studied thus far, it is clear through skin allograft studies that cytotoxic T cells can infiltrate the frog skin tissue and mediate rejection of non-self-tissue and exemplifies the conservation of adaptive immunity and allograft rejection akin to mammalian studies (32, 36, 37). In addition, B cells were also found capable of infiltrating frog skin in response to transplantation of Western clawed frog (*Xenopus tropicalis*) skin onto *X. laevis* (38).

### Glands

A hallmark of amphibian skin is the presence of varied glands located in the spongious dermal layer (Figure 1) that support the vital physiological functions performed by frog skin including, but not limited to, respiration, ion regulation, water transport, immune function and predator defence (2, 6, 9). The most ubiquitous and prominent glands in amphibian skin are mucosal glands and granular glands. Both types of glands are established in a sac-like formation surrounded by secretory cells that release granular contents and, myoepithelial cells that contract in the presence of appropriate stimuli (Figure 1) (39–43). While the precise molecular mechanisms have not yet been elucidated, whole frog studies have demonstrated that electrical stimulation, injection with norepinephrine to the dorsal lymph sacs, or chasing a frog in a bucket for 5–10 min, stimulates the release of mucosal and granular gland contents (17, 39, 40, 42, 43).

Mucosal glands secrete mucus to maintain the moisture, permeability and elasticity of the skin, all of which are necessary for amphibian homeostasis (2, 9, 44). Though species dependent, mucosal glands are generally widely distributed across frog dorsal and ventral skin, with a higher density existing on the dorsal surface (45). The pattern of mucus discharge also varies across species. In general, terrestrial and basking frogs appear to secrete mucus at a more constant rate to aid in heat exchange and water balance. Since aquatic, arboreal and nocturnal frogs do not experience the same level of evaporative water loss, the maintenance of skin moisture is more dependent on the environment (44, 46). Notable exceptions to this general observation are *X. laevis* and *X. tropicalis*; although they are largely aquatic in nature, these Sub-Saharan native frogs appear to maintain continuous mucus coverage (47–49). In accordance with this, the observation of skin in *Telmatobius* aquatic frogs showed a more even density of granular and mucosal glands between the dorsal and ventral skin, although the mucosal glands are relatively small (50). The study of mucus production in the skin remains challenging. There is difficulty in determining natural physiological parameters of mucus production such as the volume of mucus on the skin, rate of mucus production and discharge, and the ability to determine exact concentrations of skin-secreted compounds in the mucus.

Granular glands, which include small mixed glands and other types of specialized granular glands (Figure 1), have been identified in frog skin and contain bioactive molecules involved in host defence and predator defence. Granular glands, and their contents, are arguably the best studied amphibian skin gland due to the rich diversity of biomolecules they secrete–notably antimicrobial peptides and toxic alkaloids. Although commonly referred to as granular glands, these glands have the potential to secrete serous fluid, or toxic substances, and are therefore also known as serous or parotoid/venom glands (6, 41). Granular (serous) glands contain bioactive molecules with demonstrated broad-spectrum antimicrobial activity, many of which are classified as antimicrobial peptides (AMPs, discussed below; Figure 1) (6, 51, 52). Granular (parotoid/venom) glands may also sequester and release toxic alkaloid biomolecules that function in predator deterrence and/or defence (6, 53, 54). While relatively less abundant, granular glands maintain a similar distribution, and density pattern compared to mucosal glands wherein there is a higher density of granular glands on the dorsal side than the ventral side (45, 55). Granular glands appear to be further concentrated in specific regions of the skin, such as the central region of the skin vs. head or leg regions (45, 55). Similar to granular glands, small mixed glands host a reservoir of biologically active molecules or mucus and appear more evenly spread across the skin surface (29, 56). Other types of specialized glands have been identified in certain frog species with apparent functions ranging from greater granular content storage capacity, lipid secretion, and odorous secretion for predator deterrence (40, 57, 58). Skin gland diversity, both in type and chemical composition within the glands, varies with frog species and developmental stage (59–61).

## Skin as a Physical Barrier

### Cellular Junctions and Importance in Barrier Integrity

Across all vertebrates, skin is, undoubtedly, an important physical barrier between an organism and its environment. Skin barrier integrity and permeability is maintained by cellular junctions primarily between epithelial cells and include tight junctions, gap junctions, adherens junctions, and desmosomes (4, 62–64). The key proteins which comprise these junctions (and those present in mammalian skin epidermis) include claudins (claudin-1) and occludins that form tight junctions, cadherins (E-cadherin) that form adherens junctions, connexins (connexin-43) that comprise gap junctions, and desmogleins (desmoglein-3) that comprise desmosomes (62). All cellular junctions are pertinent to overall skin integrity: tight junctions connect neighbouring cells at the apical membrane, adherens junctions, and desmosomes aid in further stabilizing cell-cell adhesion, and gap junctions form channels between adjoining cells necessary for cell-cell communication (62). Tight junction proteins are detected as early as the gastrulation stage and persist until full development (4). The presence of tight junction claudin-1 proteins is crucial during gastrulation in *X. laevis* embryos (65), but general observations of tight junction proteins in adult frogs are lacking. In early larvae, tripartite junctional complexes of tight junction, adherens junction, and desmosomes are observed, wherein these complexes appear to lose significant contribution from adherens junctions in larvae approaching metamorphosis and in adult frogs (4). Nonetheless, strong expression of adherens-dependent cadherin protein has been detected in adult *X. laevis* skin (66). While the presence of gap junctions and desmosomes have been reported in the skin of other vertebrates, the observation of these junctions in frogs has been limited to observations in frog embryos undergoing development, or simple presence identification in adult frogs (67–70). Though all cellular junctions have been identified in frogs at different developmental stages, it is important to note that these studies have been limited to *X. laevis* and *R. pipiens* species and thus may not necessarily be representative of all frogs. Collectively, epithelial cell junctions allow for a continuous epithelial network that is relatively closed to the external environment while remaining open to the basal collagenous-rich dermal layers. As such, maintenance of epithelial cellular junctions is important for barrier integrity, and thus pathogen defence, particularly considering the relatively thin epidermal layer in frogs.

Skin permeability, and thus barrier integrity, is a feat made possible by cellular junctions, wherein changes in junction proteins in response to environmental conditions regulate permeability. Tight junctions are specially known to contribute to paracellular transport of molecules (i.e., through the intercellular space and across epithelium) and thus integral to epithelial permeability in mammals, fish and frogs (64, 71, 72). A plethora of studies in mammalian models describe the impact on barrier integrity, and thus barrier function, in response to various skin diseases or environmental factors (62, 71, 73). In general, downregulation of tight junction associated proteins is widely observed among an array of human skin diseases, relating to a weakening of barrier integrity (71, 73). Presence of microbes, whether commensal or pathogenic, triggers an initial upregulation of genes encoding for tight junction proteins, and thus skin barrier strengthening (71, 73). However, persistence of pathogens leads to downregulation of gene expression for junction proteins and eventual weakening of the skin barrier (71, 73). In addition to this, studies on mammalian and fish mucosal tissue, such as the gastrointestinal tract, have defined the importance of barrier integrity in response to pathogen invasion (12, 13, 15, 74). In adult frog skin, the interplay between cellular junctions and influx/efflux of water and ions demonstrates the participation of tight junctions in acting as a selective permeable interface between the frog and its environment (2, 64, 75). While the importance of the skin barrier and of the cellular junctions necessary for maintaining barrier integrity is well-reported in vertebrates (63, 73, 76), the investigations on the regulation of skin barrier integrity in adult frogs in response to environmental stimuli is lacking.

Skin sloughing, a normal process in the maintenance of amphibian skin (77, 78), may function as an innate immune barrier. Skin sloughing may serve to remove skin-associated microbes, including pathogens (79), and the rate of skin sloughing increases with certain infections, perhaps as a mechanism to limit pathogen numbers on the skin (77). However, sloughing also exposes the underlying non-keratinized layers of the skin barrier (77). The underlying mechanism controlling the rate of skin sloughing is unclear and requires further investigation.

### Mucus

Mucus plays a critical role in physical and chemical defence against pathogen invasion (12, 13, 24, 74). Recent studies observing the epidermis of *X. tropicalis* tadpoles have identified the development of multiciliated cells, ionocytes, goblet cells, and small secretory cells as integral to establishing a mucosal barrier (49, 80, 81). Manipulation of the mucus barrier composition in *X. tropicalis* tadpoles has demonstrated the key role of the Otogelin-like structural mucin glycoprotein, that provides a 6 μm thick mucosal surface barrier on tadpoles, towards conferring protection to infection of tadpoles with *Aeromonas hydrophila* (49). Presumably in conjunction with mucous, ciliated cells within the epidermal layer aid in removing trapped pathogens from the skin surface (24, 25, 49, 82). Current observation of the contribution of the skin mucus macromolecule composition in adult frogs to pathogen defence is lacking. In this regard, the mucus functions as a physical barrier. Yet, mucus also provides a framework for the various secretions from granular and small mixed glands, thereby contributing to the establishment of a formidable chemical barrier (2, 6, 44).

## Chemical barriers

### Antimicrobial Peptides

In general, antimicrobial peptides (AMPs) from metazoans are gene-encoded cationic and hydrophobic molecules ranging from 12 to 50 amino acids in length (83). AMPs have been shown to aid in the direct defence against pathogens and recent investigation has uncovered the role of AMPs in modulating immune responses in human and mouse systems (84–86). Frog skin is the most abundant natural source of AMPs found on earth (87, 88). The diversity of AMPs in frogs may not be surprising considering the biphasic life cycles of many frog species; residing in an aquatic environment during tadpole development and transitioning to a terrestrial environment post-metamorphosis. Exposure of frogs to aquatic and terrestrial pathogens, or contact with other animals that serve as pathogen reservoirs, can enhance the incidence of disease and host mortality (89, 90), necessitating the evolution of a broad arsenal of antimicrobial defence. AMPs in human skin have been extensively characterized and it is generally accepted that disruption of AMP expression may lead to cutaneous disease (91–94). Similarly, a lack of AMPs on frog skin has been shown to be detrimental to adult *X. laevis* defence against the fungal pathogen *Bd* (17). It is evident that AMPs serve a significant role in the defence of frog skin against pathogens, however our understanding of the ability of frog AMPs to exert antimicrobial activity on frog pathogens is limited and knowledge surrounding their potential immunomodulatory activity in frogs is completely lacking.

#### Structure and Diversity of Frog Skin Derived Antimicrobial Peptides

To date, 1,078 unique AMPs have been identified from amphibians (95). Collectively, amphibian AMPs are slightly shorter than mammalian AMPs, ranging from 12 to 46 amino acids (96), with no two AMPs identical in amino acid composition. Although metazoan AMPs can be classified into one of four groups based on structure alone, including alpha-helical, beta-sheet, mixed and linear, most amphibian AMPs belong to the alpha-helical and linear groups of peptides (97, 98). The major classes of frog AMPs include: brevinins, cathelicidans, dermaseptins, esculentins, japonicins, magainins, nigrocins, palustrins, ranatuerins, ranalexins, temporins, and tigerinins (99–103), although not all AMP classes are expressed in the skin of any given frog species. For example, *X. laevis* harbours four distinct families of AMPs: caerulein precursor fragment (CPF), peptide glycine-leucine-amide (PGLa), xenopsin precursor fragments (XPF) and magainins (104). In fact, the most well-characterized frog AMPs to date are of the magainin family, magainin-1 and magainin-2 (105–107). Magainin-1 and magainin-2 are both 23 amino acids and differ in the composition of 2 amino acids. Both magainins possess an alpha-helical structure and, like most AMPs, are amphipathic (105). The native structure and biochemistry of frog AMPs is particularly important as it dictates AMP function (108) and allows for intrinsic interactions with anionic membranes, such as those found on bacteria, fungi, viruses, and parasites (109). The association of the frog skin AMPs with anionic membranes, and the mechanisms by which they disrupt membrane integrity, are well-studied (110–114). The mechanisms responsible for disrupting membrane integrity are heavily influenced by lipid composition (111, 115, 116), and include lipid flip-flop, leakage, or transmembrane integration (111, 115, 117).

The distribution of AMPs across frog species is sporadic and some do not appear to synthesize AMPs at all (118). The ability to synthesize AMPs has been suggested to confer an evolutionary advantage to frogs but is not required for the survival of a species (118). For example, Coqui frogs (*Eleutherodactylus coqui*) have been shown to survive with a lack of AMPs, even when the deadly chytrid fungus, *Bd*, is detected on their skin (119). However, discovery of AMPs has traditionally relied on the isolation of active fractions from amphibian skin or amphibian skin secretions and *in vitro* testing on microbes of human importance (120, 121). Thus, there may exist additional AMPs present in amphibian skin that have previously gone unidentified (122). The use of transcriptomic approaches to investigate immune function of frog skin has yielded an effective strategy to identify AMP peptide diversity across frog species, developmental stage, and environmental factors (e.g., abiotic and biotic elements) (123–125). Recent transcriptomic approaches applied to frog skin tissues have illustrated the power of untargeted approaches to identify AMPs in frog skin and suggests the existence of a greater number and diversity of AMPs produced in individual frog species (126, 127).

AMP secretion from granular glands is constitutive and can be inducible in response to stress, injury or infection (99, 128). Although difficult to quantify the rate at which peptides are secreted, concentrations of peptides in the skin mucus of *X. laevis* has been reported at 3,256 μg/ml (constitutive secretion) whereas the average amount of AMPs found in the skin mucosal secretions of chase-stressed or norepinephrine injected *X. laevis* (inducible secretion) was 19,581 and 41,646 μg/ml, respectively (17). Both transcription and translation are likely responsible for the low levels of AMPs found on the skin of resting animals (17). However, few studies have examined the molecular mechanisms that lead to the inducible transcription of frog skin derived AMPs (129). In other organisms, such as humans, bovine and insects, the promoter regions of AMP genes have been found to harbour nuclear factor kappa-beta (NF-κB) transcription factor binding motifs and were identified as important regulatory elements for AMP gene expression (130–132). Nuclear factor kappa-beta (NF-κB) may also stimulate the transcription of AMP genes in frog skin as NF-κB has been shown to immunolocalize with the glandular cells of Chinese brown frogs (*Rana dybowskii*) (129, 133). However, nuclear localization was not apparent from these studies. In addition, NF-κB, nuclear factor NF-IL6, or cis-regulatory element 2 (CRE2) transcription binding sites have been identified in the promoter regions of several AMP genes in wrinkled frogs (*Rana rugosa*) (134), oriental fire-bellied toads (*Bombina orientalis*) (135), *X. laevis*, and *X. tropicalis* (136). Future investigation is required to dissect the potential role of NF-κB-mediated frog skin AMP gene expression, and/or other putative transcription factors, in the maintenance of frog skin homeostasis and rapid AMP production and secretion during stress, wounding, or pathogen insult.

#### Functions of Frog Skin Derived Antimicrobial Peptides

##### Direct antimicrobial activity towards frog pathogens

Extensive investigation has demonstrated frog AMPs to exert broad-spectrum antimicrobial activity against human pathogens, including bacteria, viruses, fungi and parasites, reviewed in (120, 121, 137, 138). Only recently, however, has there been a shift in focus towards understanding whether frog skin derived AMPs are antimicrobial to frog pathogens. Emerging infectious diseases continue to decimate worldwide amphibian populations and, pathogens, such as ranaviruses and *Bd*, are implicated as proximal causes in frog declines (139). It is critical to gain a further understanding of how to mitigate these diseases in order to conserve dwindling frog populations.

Frog skin derived peptides that have been tested for anti-pathogen activity span a diverse range of peptide families from several frog species and collectively have anti-bacterial (Tables 1, 2), anti-viral (Table 3), anti-fungal (Table 4), and anti-parasite (Table 5) activities. Frog AMPs are effective antimicrobial agents against *Aeromonas* sp., the causative agents of red-leg, a polymicrobial disease that is characterized by congestion of the skin, ulceration, haemorrhage, bloating, failure to respond to stimuli, and tetanic seizures (150). Differences in susceptibility to AMPs exist across *Aeromonas* sp. and illustrate that there exists some microbial selectivity to antimicrobial action. For example, *Aeromonas caviae* are highly susceptible to dermaseptin-S1 from the waxy monkey tree frog (*Phyllomedusa sauvagii*) with minimal inhibitory concentrations (MICs) as low as 0.5–1 μM, while other *Aeromonas* strains such as *A. hydrophila* have been reported to be resistant to dermaseptin-S1 (Table 1). In addition, some bacteria appear to be completely refractory to antimicrobial peptide families. For example, AMPs (either single AMPs or mixed preparations) from *X. laevis* failed to inhibit *A. hydrophila* growth (Tables 1, 2). However, peptides from *X. laevis* were very effective against *Citrobacter fruendii*, another causative agent of red-leg, either alone or in combination (Tables 1, 2). In addition, magainin-2 alone was not effective against *Chryseobacterium meningiosepticum* but when the natural mixture of *X. laevis* skin secretions was applied to this pathogen, it was effective at reducing its growth (Tables 1, 2). This evidence suggests that some peptides may require synergy to work against select pathogens.

**Table 1:** Minimal inhibitory concentration (MIC) of individual frog skin-derived antimicrobial peptides against amphibian bacterial pathogens.

**Pathogen**	**Species**	**AMP**	**Sequence**	**MIC**	**References**
*Aeromonas caviae*	*Phyllomedusa sauvagii*	Dermaseptin-S1	ALWKTMLKKLGTMALHAGKAALGAAADTISQGTQ	0.5	(140)
		Dermaseptin-S2	ALWFTMLKKLGTMALHAGKAALGAAANTISQGTQ	1	(140)
		Dermaseptin-S3	ALWKNMLKGIGKLAGKAALGAVKKLVGAES	1	(140)
		Dermaseptin-S4	ALWMTLLKKVLKAAAKAALNAVLVGANA	0.5	(140)
		Dermaseptin-S5	GLWSKIKTAGKSVAKAAAKAAVKAVTNAV	35	(140)
*Aeromonas hydrophila*	*Litoria ewingii*	Aurein 2.1	GLLDIVKKVVGAFGSL	ND	(141)
	*Phyllomedusa sauvagii*	Dermaseptin-S1	ALWKTMLKKLGTMALHAGKAALGAAADTISQGTQ	ND	(142)
		Ranalexin	FLGGLIKIVPAMICAVTKKC	ND	(142)
	*Rana catesbeiana*	CPF	GFASFLGKALKAALKIGANLLGGTPQQ-OH	ND	(142)
	*Xenopus laevis*	Magainin I	GIGKFLHSAGKFGKAFVGEIMKS	ND	(142)
		Magainin II	GIGKFLHSAKKFGKAFVGEIMNS	ND	(142)
		Magainin II	GIGKFLHSAKKFGKAFVGEIMNS	ND	(141)
		PGLa	GMASKAGAIAGKIAKVALKAL.NH2	ND	(142)
*Citrobacter freundii*	*Ascaphus truei*	Ascaphin-8	GFKDLLKGAAKALVKTVLF.NH2	6	(143)
	*Litoria ewingii*	Aurein 2.1	GLLDIVKKVVGAFGSL	100	(141)
	*Leptodactylus pentadactylus*	Leptoglycin	GLLGGLLGPLLGGGGGGGGGLL	75	(144)
	*Xenopus laevis*	Magainin II	GIGKFLHSAKKFGKAFVGEIMNS	30 μg/ml	(99)
		Magainin II	GIGKFLHSAKKFGKAFVGEIMNS	50	(141)
*Chryseobacterium meningiosepticum*	*Litoria ewingii*	Aurein 2.1	GLLDIVKKVVGAFGSL	ND	(141)
	*Xenopus laevis*	Magainin II	GIGKFLHSAKKFGKAFVGEIMNS	ND	(141)
*Klebsiella pneumoniae*	*Litoria ewingii*	Aurein 2.1	GLLDIVKKVVGAFGSL	100	(141)
	*Xenopus laevis*	Magainin II	GIGKFLHSAKKFGKAFVGEIMNS	50	(141)
*Lactococcus lactis*	*Litoria ewingii*	Aurein 2.1	GLLDIVKKVVGAFGSL	100	(141)
	*Xenopus laevis*	Magainin II	GIGKFLHSAKKFGKAFVGEIMNS	100	(141)
*Pseudomonas aeruginosa*	*Litoria ewingii*	Aurein 2.1	GLLDIVKKVVGAFGSL	200	(141)
	*Xenopus laevis*	Magainin II	GIGKFLHSAKKFGKAFVGEIMNS	50	(141)
*Proteus mirabilis*	*Litoria ewingii*	Aurein 2.1	GLLDIVKKVVGAFGSL	ND	(141)
	*Xenopus laevis*	Magainin II	GIGKFLHSAKKFGKAFVGEIMNS	ND	(141)
*Serratia liquefaciens*	*Litoria ewingii*	Aurein 2.1	GLLDIVKKVVGAFGSL	100	(141)
	*Xenopus laevis*	Magainin II	GIGKFLHSAKKFGKAFVGEIMNS	100	(141)

*ND indicates not determined (i.e., at the level tested, the AMP was found to have no effect on the pathogen). MIC values are reported in μM unless indicated otherwise*.

**Table 2:** Minimal inhibitory concentration (MIC) of skin secretions containing frog skin-derived antimicrobial peptides against amphibian bacterial pathogens.

**Pathogen**	**Species**	**Peptide Mixture**	**MIC**	**References**
*Aeromonas hydrophila*	*Litoria aurea*	Aurein 1.1, Aurein 3.1, Aurein 3.2, Aurein, 5.2, Aurein 3.3, Aurein 2.5, Aurein 1.2, Aurein 2.1, Aurein 2.6	ND	(141)
	*Litoria ewingii*	Uperin 7.1	ND	(141)
	*Litoria raniformis*	Aurein 3.1, Aurein 5.2, Aurein 3.2, Aurein 2.5, Aurein 2.1, Aurein 2.2, Aurein 2.3, Aurein 2.4	ND	(141)
	*Xenopus laevis*	Magainin I, Magainin II, PGQ, CPF 1-4, XPF, LPF, PGLa	ND	(141)
*Citrobacter freundii*	*Litoria aurea*	Aurein 1.1, Aurein 3.1, Aurein 3.2, Aurein, 5.2, Aurein 3.3, Aurein 2.5, Aurein 1.2, Aurein 2.1, Aurein 2.6	62.5 μg/ml	(141)
	*Litoria ewingii*	Uperin 7.1	ND	(141)
	*Litoria raniformis*	Aurein 3.1, Aurein 5.2, Aurein 3.2, Aurein 2.5, Aurein 2.1, Aurein 2.2, Aurein 2.3, Aurein 2.4	62.5 μg/ml	(141)
	*Xenopus laevis*	Magainin I, Magainin II, PGQ, CPF 1-4, XPF, LPF, PGLa	31.25 μg/ml	(141)
*Chryseobacterium meningosepticum*	*Litoria aurea*	Aurein 1.1, Aurein 3.1, Aurein 3.2, Aurein, 5.2, Aurein 3.3, Aurein 2.5, Aurein 1.2, Aurein 2.1, Aurein 2.6	ND	(141)
	*Litoria ewingii*	Uperin 7.1	ND	(141)
	*Litoria raniformis*	Aurein 3.1, Aurein 5.2, Aurein 3.2, Aurein 2.5, Aurein 2.1, Aurein 2.2, Aurein 2.3, Aurein 2.4	ND	(141)
	*Xenopus laevis*	Magainin I, Magainin II, PGQ, CPF 1-4, XPF, LPF, PGLa	500 μg/ml	(141)
*Klebsiella pneumoniae*	*Litoria aurea*	Aurein 1.1, Aurein 3.1, Aurein 3.2, Aurein, 5.2, Aurein 3.3, Aurein 2.5, Aurein 1.2, Aurein 2.1, Aurein 2.6	250 μg/ml	(141)
	*Litoria ewingii*	Uperin 7.1	ND	(141)
	*Litoria raniformis*	Aurein 3.1, Aurein 5.2, Aurein 3.2, Aurein 2.5, Aurein 2.1, Aurein 2.2, Aurein 2.3, Aurein 2.4	250 μg/ml	(141)
	*Xenopus laevis*	Magainin I, Magainin II, PGQ, CPF 1-4, XPF, LPF, PGLa	125 μg/ml	(141)
*Lactococcus lactis*	*Litoria aurea*	Aurein 1.1, Aurein 3.1, Aurein 3.2, Aurein, 5.2, Aurein 3.3, Aurein 2.5, Aurein 1.2, Aurein 2.1, Aurein 2.6	500 μg/ml	(141)
	*Litoria ewingii*	Uperin 7.1	ND	(141)
	*Litoria raniformis*	Aurein 3.1, Aurein 5.2, Aurein 3.2, Aurein 2.5, Aurein 2.1, Aurein 2.2, Aurein 2.3, Aurein 2.4	500 μg/ml	(141)
	*Xenopus laevis*	Magainin I, Magainin II, PGQ, CPF 1-4, XPF, LPF, PGLa	500 μg/ml	(141)
*Pseudomonas aeruginosa*	*Litoria aurea*	Aurein 1.1, Aurein 3.1, Aurein 3.2, Aurein, 5.2, Aurein 3.3, Aurein 2.5, Aurein 1.2, Aurein 2.1, Aurein 2.6	125 μg/ml	(141)
	*Litoria ewingii*	Uperin 7.1	ND	(141)
	*Litoria raniformis*	Aurein 3.1, Aurein 5.2, Aurein 3.2, Aurein 2.5, Aurein 2.1, Aurein 2.2, Aurein 2.3, Aurein 2.4	125 μg/ml	(141)
	*Xenopus laevis*	Magainin I, Magainin II, PGQ, CPF 1-4, XPF, LPF, PGLa	62.5 μg/ml	(141)
*Proteus mirabilis*	*Litoria aurea*	Aurein 1.1, Aurein 3.1, Aurein 3.2, Aurein, 5.2, Aurein 3.3, Aurein 2.5, Aurein 1.2, Aurein 2.1, Aurein 2.6	ND	(141)
	*Litoria ewingii*	Uperin 7.1	ND	(141)
	*Litoria raniformis*	Aurein 3.1, Aurein 5.2, Aurein 3.2, Aurein 2.5, Aurein 2.1, Aurein 2.2, Aurein 2.3, Aurein 2.4	ND	(141)
	*Xenopus laevis*	Magainin I, Magainin II, PGQ, CPF 1-4, XPF, LPF, PGLa	ND	(141)
*Serratia liquefaciens*	*Litoria aurea*	Aurein 1.1, Aurein 3.1, Aurein 3.2, Aurein, 5.2, Aurein 3.3, Aurein 2.5, Aurein 1.2, Aurein 2.1, Aurein 2.6	ND	(141)
	*Litoria ewingii*	Uperin 7.1	ND	(141)
	*Litoria raniformis*	Aurein 3.1, Aurein 5.2, Aurein 3.2, Aurein 2.5, Aurein 2.1, Aurein 2.2, Aurein 2.3, Aurein 2.4	ND	(141)
	*Xenopus laevis*	Magainin I, Magainin II, PGQ, CPF 1-4, XPF, LPF, PGLa	ND	(141)

*ND indicates not determined (i.e., at the level tested, the AMP was found to have no effect on the pathogen). MIC values are reported in μM unless indicated otherwise. For peptide mixtures, the antimicrobial peptides identified through mass spectrometry are summarized*.

**Table 3:** Inhibitory concentration 50 (IC_50_) of frog skin-derived antimicrobial peptides against amphibian viral pathogens.

**Virus**	**Species**	**AMP**	**Sequence**	**IC_**50**_(μM)**	**References**
*Frog virus 3*	*Phyllomedusa sauvagii*	Dermaseptin-S1	ALWKTMLKKLGTMALHAGKAALGAAADTISQGTQ	12	(10)
	*Rana catesbeiana*	Skin peptide mixture		ND	(10)
	*Rana pipiens*	Skin peptide mixture		ND	(10)
	*Rana temporaria*	Temporin A	FLPLIGRVLSGIL.NH2	58	(10)
	*Xenopus laevis*	Magainin II	GIGKFLHSAKKFGKAFVGEIMNS	ND	(10)
		PGLa	GMASKAGAIAGKIAKVALKAL.NH2	ND	(10)

*ND indicates not determined (i.e., at the level tested, the AMP or skin peptide mixture was found to have no effect on the pathogen). Values are reported in μM where available*.

**Table 4:** Minimal inhibitory concentration (MIC) of frog skin-derived antimicrobial peptides against amphibian fungal pathogens.

**Pathogen**	**Species**	**AMP**	**Sequence**	**MIC (μM)**	**References**
*Batrachochytrium dendrobatidis*	*Hylomantis lemur*	Phylloseptin-L1	LLGMIPLAISAISALSKL	100	(145)
	*Phyllomedusa sauvagii*	Dermaseptin-S1	ALWKTMLKKLGTMALHAGKAALGAAADTISQGTQ	23	(142)
	*Rana boylii*	Brevinin-1BYa	FLPILASLAAKFGPKLFCLVTKKC	12.5	(146)
		Brevinin-1BYc	FLPILASLAAKLGPKLFCLVTKKC	6	(146)
		Ranatuerin-2BYa	GILSTFKGLAKGVAKDLAGNLL DKFKCKITGC	25	(146)
		Ranatuerin-2BYb	GIMDSVKGLAKNLAGKLLDSLKCKITGC	12.5	(146)
	*Rana boylii*	Metamorph AMP mixture		12.5−50 μg/ml	(146)
	*Rana catesbeiana*	Ranalexin	FLGGLIKIVPAMICAVTKKC	9	(142)
	*Rana muscosa*	Ranatuerin-2Ma	GLLSSFKGVAKGVAKNLAGKLLEKLKCKITGC	50	(147)
		Ranatuerin-2Mb	GIMDSVKGVAKNLAAKLLEKLKCKITGC	25	(147)
		Temporin-1M	FLPIVGKLLSGLL.NH2	100	(147)
	*Rana mucosa*	Adult AMP mixture	Temporin 1-M, Ranatuerin-2Mb, and Ranateurin-2Ma	>250 μg/ml	(147)
	*Rana pretiosa*	Brevinin-1PRa	FLPVLTGLTPSIVPKLVCLLTKKC	50	(148)
		Brevinin-1PRb	FLPVLAGLTPSIVPKLVCLLTKKC	12.5	(148)
		Brevinin-1PRc	FFPMLAGVAARVVPKVICLITKKC	6.25	(148)
		Brevinin-1PRd	FLPMLAGLAASMVPKLVCLITKKC	12.5	(148)
		Esculentin-2PRa	GVFSFLKTGAKLLGSTLLKMAGKAGAEHLACKATNQC	25	(148)
		Esulentin-2PRb	GIFSALAAGVKLLGNTLFKMAGKAGAEHLACKATNQC	12.5	(148)
		Ranatuerin-2PRa	GILDSFKGVAKGVAKDLAGKLLDKLKCKITGC	25	(148)
		Rantuerin-2PRb	GILDTFKGVAKGVAKDLAVHMLENLKCKMTGC	50	(148)
		Rantuerin-2PRc	GILDSFKDVAKGVATHLLNMAKCKMTGC	100	(148)
		Rantuerin-2PRe	GIMNTVKDVATGVATHLLNMVKCKITGC	100	(148)
		Rantuerin-2PRf	GILDTFKGVAKGVAKDLAVHMLEKLKCKMTGC	25	(148)
		Rantuerin-2PRg	GILSSFKDVAKGVAKNVAAQLLDKLKCKITGC	50	(148)
		Rantuerin-2PRh	GILDTVKGVAKDVAAHLLNMVKCKITGC	50	(148)
		Temporin-PRb	FLPIITNLLGKLL.NH2	100	(148)
		Temporin-PRc	NFLDTLINLAKKFI.NH2	25	(148)
		Temporin-PRe	FLPLAMALGKLL.NH2	>100	(148)
	*Xenopus laevis*	CPF	GFASFLGKALKAALKIGANLLGGTPQQ-OH	12.5	(149)
		CPF	GFASFLGKALKAALKIGANLLGGTPQQ-OH	3.1	(142)
		Magainin I	GIGKFLHSAGKFGKAFVGEIMKS	50	(142)
		Magainin II	GIGKFLHSAKKFGKAFVGEIMNS	162	(149)
		Magainin II	GIGKFLHSAKKFGKAFVGEIMNS	100	(142)
		PGLa	GMASKAGAIAGKIAKVALKAL.NH2	50	(149)
		PGLa	GMASKAGAIAGKIAKVALKAL.NH2	3.1	(142)
		Magainin II + PGLa (1:1 ratio)		12.5	(142)
*Basidiobolus ranarum*	*Xenopus laevis*	Magainin II	GIGKFLHSAKKFGKAFVGEIMNS	12.5	(142)
		PGLa	GMASKAGAIAGKIAKVALKAL.NH2	3.1	(142)
		Magainin II + PGLa (1:1 ratio)		0.8	(142)

*MIC values are reported in μM unless indicated otherwise. For peptide mixtures, the antimicrobial peptides identified through mass spectrometry are summarized*.

**Table 5:** Minimal inhibitory concentration (MIC) of frog skin-derived antimicrobial peptides against amphibian parasites.

**Parasite**	**Frog species**	**Antimicrobial peptide**	**MIC (μg/ml)**	**References**
*Manodistomum*	*Rana catesbeiana*	Adult AMP mixture	65	(11)
*Echinostoma*	*Rana catesbeiana*	Adult AMP mixture	58	(11)
*Ribeiroia*	*Rana catesbeiana*	Adult AMP mixture	35	(11)
*Armatae*	*Rana catesbeiana*	Adult AMP mixture	20	(11)
*Alaria*	*Rana catesbeiana*	Adult AMP mixture	19	(11)

Currently, the only viral pathogen of frogs that skin AMPs have been tested on is FV3. Frog skin AMPs have mixed antiviral efficacy on FV3. While dermaseptin-S1 from the waxy monkey tree frog, (*P. sauvagii*) and temporin A from the common frog (*R. temporaria*) were capable of inactivating FV3, magainin-2 from *X. laevis* was not able to inhibit FV3 infectivity at the AMP concentrations tested (10). The synergistic activity of AMPs towards FV3 is unknown.

Not surprisingly, the majority of frog skin derived AMPs tested against fungal pathogens of frogs have focused on *Bd* (Table 4). Based on the MICs reported, the most effective anti-fungal frog skin AMPs belong to *X. laevis* and Ranid species, the foothill yellow-legged frog (*Rana boylii*) and the Oregon spotted frog (*Rana pretiosa*) (Table 4). The promising effects of frog skin AMPs have been shown be effective against *Bd* zoospores *in vitro* (151, 152) and important in *X. laevis* skin defence against *Bd* in *in vivo* infection studies (17). Although magainin-2 and PGLa applied individually to *Bd* and *Basidiobolus ranarum*, (another fungus that infects the skin of amphibians) were quite effective at reducing fungal growth, the combination highly reduced the MIC required to inhibit the fungi (i.e., was more potent) (Table 4). This is strong evidence to support that synergistic mechanisms may be more beneficial in combating particular pathogens than individual peptides. In general, the minimal inhibitory concentration of frog skin AMPs required to inhibit fungal pathogens is much higher than the amount required to inhibit bacteria or viruses (Table 4).

There is also some evidence to support the anti-parasitic role of frog skin AMPs (11). A native mixture of peptides obtained from adult *R. catesbaeiana* skin was effective at inhibiting trematode cercariae viability at all AMP mixture concentrations tested (Table 5). However, the peptide composition was not determined. To date, 12 different AMPs have been identified in *R. catesbeiana* skin secretions (95). Albeit limited in number, these studies demonstrate frog skin AMPs to be direct antimicrobial agents in innate immune defence against frog pathogens.

##### Wound healing

Research in murine models demonstrate that mammalian AMPs such as cathelicidan-related antimicrobial peptide are beneficial in combating skin infections in mice where they clear invading bacteria, activate immune cells and promote wound closure (78, 153–155). In mammalian systems, AMPs can bind cell surface receptors such as formyl peptide receptor-like-1 (FPLR1), purinergic receptors (P2X_7_), Toll-like receptors (TLRs), chemokine receptors (CCRs), G-protein coupled receptors (GPCRs), and epidermal growth factor receptor (EGFR) to activate downstream signalling pathways to promote wound healing (156). A few studies have examined the ability of frog skin AMPs to promote wound healing in mammalian models. The application of cathelicidan-NV from the skin of a plateau frog (*Nanorana ventripunctata*) onto wounded mouse skin resulted in the acceleration of wound re-epithelization by direct stimulation of keratinocyte motility and proliferation (157). Cathelicidan-NV treatment also upregulated numerous genes involved in migration, proliferation and differentiation in wounded mouse skin tissue (157). Another frog skin AMP, esculentin-1a(1-21) from the common European frog (*Rana esculenta*), also promoted wound healing by stimulating human keratinocyte migration (158). Although frog skin AMPs are capable of promoting wound healing in mouse systems, and frogs are known for their remarkable wound healing ability, the function of frog skin AMPs in frog skin wound healing and the underlying cellular and molecular mechanisms are still unclear.

##### Immunomodulation of innate immunity

The most well-characterized human AMPs belong to the cathelicidan (LL-37) and defensin (hBD-1, hBD-2, hBD-3, hBD-4, HD-5, and HD-6) families (156, 159, 160) and are also considered host defence peptides (HDPs) since they have been shown to modulate innate and adaptive immune responses of homologous host cells (156, 161). Treatment of mammalian cells with frog skin AMPs (i.e., heterologous system) revealed mammalian cells to be responsive to frog skin AMPs (157, 158, 162, 163). For example, Esculentin-1a(1-21) treatment of human keratinocytes resulted in increased phosphorylation of signal transducer and activator of transcription 3 (STAT3), activating the transcription of downstream genes involved in wound healing (158). Cathelicidan-NV induced fibroblast-to-myofibroblast transition and also significantly increased collagen production in the wound (157). Another frog skin AMP, brevinins-1Pa, from *R. pipiens*, stimulated the release of insulin from rat pancreatic islet cells (162). Insulin is known to play a role in keratinocyte function by inducing migration through the PI3-Akt-RhoA network (164). Several AMPs from *X. laevis* (CPF, magainin-1, magainin-2, PGLa) and the Taiwanese frog (*Holobatrachus rugulosas*) tigerinin-1R have been shown to stimulate the secretion of glucagon-like peptide 1 (GLP-1) from GLUTag cells (163). GLP-1 is an immunomodulatory molecule and decreases the inflammatory response during allergen and infection-induced inflammation (165). The experimental evidence suggests that various frog skin AMPs have a substantial effect on mammalian cells processes such as cell migration, inflammation, immunity and repair (158, 164, 166). Unfortunately, the functions of frog skin AMPs on frog cells (i.e., homologous system) have not been explored and whether frog skin AMPs act as HDPs in frogs remains unknown.

### Alkaloids

Lipid-soluble alkaloid compounds are believed to originate from the amphibian diet, largely from insects (167). Identification of these alkaloid compounds has mainly focused on those excreted by frogs in the Dendrobatidae family (poison dart frogs) with observation from over 150 species (168, 169). Nonetheless, toxic alkaloid substances have also been observed in Eleutherodactylidae, Leptodactylidae, Mantella, Myobatrachidae, and Ranidae frogs (54, 169–171). Toxic alkaloids are primarily involved in predation avoidance, however, a few also participate in defence against microbes (167, 172). Readers interested in alkaloid compound diversity are referred to reviews on this topic (169, 173).

## Epithelial Cells as Microbial Sensors and Initiators of Innate Immune Responses

Epithelial cells are emerging as crucial contributors to innate immune responses through the detection of microorganisms–both commensal and pathogenic—in the external environment (174, 175) through the use of pattern recognition molecules. A relatively limited number of germ-line encoded pattern recognition receptors (PRRs) detect non-self and damage signals and these recognition events are crucial to initiating innate immune response. Classes of PRRs are generally divided into transmembrane and cytosolic PRRs. Transmembrane receptors include TLRs, C-type-lectin like receptors (CLRs), and scavenger receptors, while cytosolic PRRs include retinoic acid inducible gene- (RIG-) I-like receptors (RLRs), nucleotide-binding oligomerization domain- (NOD-) like receptors (NLRs), and various cytosolic DNA sensors (176). Collectively, PRRs recognize a variety of pathogen-associated molecular patterns (PAMPs), also known as microbial-associated molecular patterns (MAMPs), including lipopolysaccharide, peptidoglycan, lipopeptides, flagellin, single stranded RNA, double stranded RNA, double stranded DNA, carbohydrate structures, as well as other PAMPs (176). PRRs also recognize damage-associated molecular patterns (DAMPs) released upon cellular stress (177). Ligand sensing by PPRs leads to intracellular signalling cascades that regulate the transcription of genes encoding for pro-inflammatory, chemoattractive and anti-viral functions (176). In accordance with the location of epithelial cells at the host-environment interface, epithelial cells in mammalian models have been shown to express diverse PRRs including TLRs (178), RLRs (179), and NLRs (180) to sense invading microorganisms and initiate innate immune responses.

Few studies have focused on the characterization of amphibian PRRs (e.g., ligands, signalling pathways, downstream gene targets), let alone their role in amphibian skin epithelial cell biology. Yet, it is evident that cells within frog skin tissue are capable of sensing bacterial, viral and fungal pathogens, including commercially available mimics of PAMPs, and initiate innate immune responses through the upregulation genes encoding for pro-inflammatory cytokines, anti-viral cytokines, antimicrobial peptides, and other immune proteins (181–183). The cell type(s) and receptors involved in microbial recognition by amphibian skin tissues are largely unknown. Thus, much of our basis for understanding the role of frog skin epithelial cells to microbial detection is limited to the identification of key pattern recognition molecules in the frog genome and implied conservation of their function based on limited expression data in frog skin tissues. In the below subsections, we summarize the current state of knowledge surrounding the presence of genes encoding for pattern recognition molecules identified in frog genomes and the expressions of these genes in frog skin tissues.

### Toll-Like Receptors (TLRs)

The first glimpse into the frog TLR multigene family came about through a bioinformatics approach to study the evolution of vertebrate TLRs and was spearheaded as a result of the influx of draft genome sequences of fish (e.g., *Takifugu rubripes)* and frog (*X. tropicalis*) (184). Molecular evolutionary analysis demonstrated that TLRs are evolving at approximately the same, slow rate and are under strong purifying selection, presumably to ensure maintenance of TLR function both in terms of ligand recognition and initiation of intracellular signalling cascades (184, 185). Through the construction of molecular trees, six major TLR families emerged (Table 6), each encompassing subfamilies of TLRs that that recognized a general set of PAMPs/MAMPs (184, 186). At least 19 TLR genes were identified in the *X. tropicalis* genome (JGI 4.1) and included orthologues of both mammalian and fish specific (e.g., TLR21, TLR22) TLRs. Characteristic of the mammalian TLR2 family is the ability of TLR2 family members to form heterodimeric pairs with TLR2 (176) to recognize a diverse set of ligands, and is presumed to also occur in frogs (184). In *X. tropicalis* the TLR2 family encompasses one TLR1, two TLR2, two TLR6, and four TLR14 subfamily members and appears to lack the TLR10 subfamily (Table 6) (184, 186). The TLR14 subfamily appears to have expanded in *X. tropicalis*, and possibly in other frogs, to four TLR14 subfamily members that are hypothesized to form heterodimeric pairs with TLR2, similar to other subfamily members of the TLR2 family (184). One member of each of the TLR3 (senses dsRNA), TLR4 (senses LPS) and TLR5 (senses flagellin) families were identified in *X. tropicalis* (Table 6) (184, 186). However, the putative *X. tropicalis tlr4* gene does not appear to encode for a transmembrane region based on *in silico* structural prediction (186). Genes for *cd14* or *md-2*, involved in TLR4 function in mammals (176), have not been identified in the *X. tropicalis* genome and thus the function of the putative *X. tropicalis* TLR4 as an LPS sensor is uncertain (186). Another interesting deviation from the mammalian system is the predicted presence of a soluble TLR5, termed *tlr5s* (184), similar to the soluble TLRs found in fish species (188). The *tlr5s* gene is predicted to encode for the extracellular leucine rich repeat (LRR) region and is lacking the transmembrane and intracellular TIR signalling domains suggesting it may act as a soluble receptor to potentially regulate TLR5 signalling (184). The TLR7 family is crucial for sensing endosomal PAMPs in mammals (189) and a single orthologue of *tlr7* and *tlr9*, and *two* orthologues of *tlr8* were identified in *X. tropicalis* (Table 6) (184, 186). Lastly, a single orthologue of TLR12, TLR13, TLR21 and TLR22 subfamilies were identified in *X. tropicalis* (Table 6) (184, 186). *In silico* prediction of *X. tropicalis* TLRs protein structures revealed overall similar *X. tropicalis* TLR structure to corresponding human TLR orthologues, including a similar size and number of LRR domains, transmembrane region and an intracellular TIR domain (186).

**Table 6:** Toll-like receptor genes identified in frog species.

**Family**	**Subfamily**	***X. tropicalis***	***X. laevis***	**Other frogs**
**TLR2**	TLR1	*tlr1* (186)	*tlr1*^$^ (186)	*B. maxima*^$^ (183)
	TLR2	*tlr2.1, tlr2.2* (186)	*tlr2*^$^ (186)	*R. japonica*^$^(187) *R. ornativentris*^$^ (187) *R. tagoi tagoi*^$^ (187) *B. maxima*^$^ (183)
	TLR6	*tlr6.1, tlr6.2* (186)	*tlr6*^$^ (186)	*B. maxima*^$^ (183)
	TLR10	Not identified (184, 186)		
	TLR14 and TLR14-like	***tlr14.1**, tlr14.2, tlr14.3, tlr14.4* (184, 186)	*tlr14*^$^ (186)	
**TLR3**	TLR3	***tlr3*** (184, 186)	*tlr3*^$^ (186)	*B. maxima*^$^ (183)
**TLR4**	TLR4	*tlr4* (184) *tlr4* found in non-coding region (186)	*tlr4*^$^ (186)	*R. japonica*^$^(187) *R. ornativentris*^$^ (187) *R. tagoi tagoi*^$^ (187) *B. maxima*^$^ (183)
**TLR5**	TLR5	***tlr5*** (184, 186), tlrs5^*^ (184)	*tlr5*^$^ (184, 186)	*B. maxima*^$^ (183)
**TLR7**	TLR7	*tlr7* (184, 186)	*tlr7*^$^ (186)	*B. maxima*^$^ (183)
	TLR8	***tlr8.1**, tlr8.2* (186)	*tlr8*^$^ (186)	*B. maxima*^$^ (183)
	TLR9	*tlr9* (184, 186)	*tlr9*^$^ (186)	
**TLR12**	TLR12	*tlr12* (186)	*tlr12*^$^ (186)	
	TLR13	***tlr13*** (184, 186)	*tlr13*^$^ (186)	
	TLR21	*tlr21* (184, 186)	*tlr21*^$^ (186)	
	TLR22	*tlr22* (184, 186)	*tlr22*^$^ (186)	

X. tropicalis tlr genes shown in bold are predicted to contain introns, non-bolded genes are predicted to be intronless.

* Soluble short form lacks the transmembrane and TIR domains.

$ *Sequences were detected by RT-PCR with cDNA as a template, gene sequence structure not reported*.

Aside from the identification of TLR genes in few frog species (125, 183, 184, 186), little investigation has focused on characterization of frog TLRs, and their role in frog skin innate immunity. In *X. laevis*, the TLR genes, including the putative *tlr4*, are expressed in the skin of tadpoles and adults (181, 186). Transcriptomic studies from skin of healthy Japanese brown frogs (*Rana japonica*), Montane brown frog (*Rana ornativentris*), Tago frog (*Rana tagoi*) (187), and the Yunnan firebelly toad (*Bombina maxima*) (183) have demonstrated the presence of *tlr* transcripts in skin tissue and further support the important role of anuran skin and the cells within as important sensors of microbes and regulators of innate immunity. Indeed, several transcriptomic studies of anuran skin tissues, including *Ranidae, Megophryidae, Rhacophoridae*, and *Bufonidae* families, revealed the enrichment of transcripts involved in processes reflected in the gene ontology terms “immune system process,” “immune system,” and “signal transduction,” further supporting anuran skin as an immune organ (123–125). However, only a single study has examined the potential sensing of a PAMP by a frog TLR; LPS (10 μg/ml) treatment of *R. temporaria* frog urinary bladder epithelial cells positive for TLR4 (albeit demonstrated through the use of non-homologous anti-TLR4 antibody) triggered epithelial cell activation through an NF-κB dependent mechanism (190). Although these urinary bladder epithelial cells appear to be LPS responsive, unequivocal evidence that TLR4 is responsible for LPS sensing is lacking.

### Cytosolic Pattern Recognition Sensors

RLRs, NLRs, and cytoplasmic DNA sensors are vital cytosolic pattern recognition molecules involved in initiating pro-inflammatory and anti-viral responses (191). RLR family members include retinoic acid-inducible gene-I (RIG-I), melanoma differentiation-associated gene 5 (MDA5), and laboratory of genetics and physiology 2 (LGP2) (191). In mammals, RIG-I and MDA5 bind viral RNA via the common RNA helicase domain and ligand recognition results in activation of interferon regulatory factor 3 and NF-kB transcription factors to initiate transcription of an anti-viral interferon response (191). LGP2 is known to interfere with viral RNA binding to RIG-I and MDA5 (192). While *rig-i, mda5*, and *lgp2* genes have been identified in the *X. tropicalis* genome (193) and *rig-i* and *mda5* found expressed in frog skin (181, 183), little else is known about the role of RLRs in anurans.

In mammals, NLRs are organized into five subfamilies (NLRA, NLRB, NLRC, NLRX, NLRP) based on the N-terminal effector domain and collectively sense a wide range of MAMPs (194). NLR activation leads to receptor oligomerization and formation of the inflammasome and activation of downstream inflammatory caspases that cleave interleukin 1 cytokine family members (IL-1, IL-18) (194). Seven NLR genes were identified in the *X. tropicalis* genome, including NLRA/CIITA, NLRC1/NOD1, NLRC3, NLRC4, NLRC5, and NLRX1, while NLRC2/NOD2 appears to be absent (195, 196). Members of all five NLR subfamily were identified in the *B. maxima* skin transcriptome including NLRA/CIITA, NLRB/NAIP, NLRC1/NOD1, NLRC3, NLRC5, NLRP1, NLRP3, NLRP5, and NLRX1 (183).

In addition to RLRs and NLRs, cytosolic DNA sensors are also expressed in frog skin. Amphibian skin transcriptomes from the Chinese giant salamander (*Andrias davidianus*), Asiatic toad (*Bufo gargarizans*), and black-spotted frog (*Rana nigromaculata*) revealed the presence of transcripts in the “cytosolic DNA-sensing pathway” and the expression of a DNA-dependent RNA polymerase III that functions as a cytosolic DNA sensor by transcribing an RNA copy for recognition by RIG-I (124), suggesting a conserved evolutionary anti-microbial mechanism. However, an AIM-2-like receptor, another cytosolic DNA sensor that can lead to inflammasome activation, is seemingly absent in *X. tropicalis* (195).

## Impact of Environment on Host Barriers

### Abiotic Factors

Frogs are to the environment as canaries were to coal mines. They are an important indicator species and their physiology is heavily influenced by the environment (197–199). Most studies examining the impact of abiotic factors on amphibian skin have focused on AMPs. Temperature, dehydration, shade, acidification, oxygen and altitude (200, 201) have been documented to influence frog skin AMPs. For example, increased environmental temperatures (from 5 to 30°C) triggered brevinin-1SY AMP production in *R. sylvatica* skin tissue (200). Albeit, the underlying mechanism for the production of brevinin-1SY at higher temperatures is unclear, increased microbial colonization of the skin at the higher temperature or increased transcriptional/translational kinetics may be involved. Microbes on the skin surface may stimulate PRRs on the membrane of epidermal cells, leading to downstream signalling that potentially induces transcription of AMP genes with NF-κB in the promoter region (202). Cold stress in mammals (203) and cultured amphibian primary epidermal cells (204) has been shown to reduce the rates of transcription and translation, leading to decreased global protein synthesis. Thus, low body temperatures of *R. sylvatica* may have led to a near halt in AMP synthesis. In *R. catesbeiana* tadpoles, shade and acidification of the environment have been shown to modulate the production and bioactivity of AMPs (201, 205). Another environmental factor that has an effect on AMPs is hydration status. Dehydration in *R. sylvatica* increased the expression of brevinin-1SY in the skin (201). In addition to dehydration, other environmental stressors such as anoxia or freezing, also enhances the antimicrobial activity of *R. sylvatica* brevinin-1SY against select microbial strains (201). Decreased oxygen availability or hypoxia, has been associated with an increased number of granular glands in Tibetan frog (*Nanorana parkeri*) middorsal skin (199). The biological significance of increased granular glands found in hypoxic conditions is unknown. It is evident from these findings that the regulation of AMPs and the diversity among the AMP secretome is complex but is shaped by the environment.

### Chemical Contaminants

Anthropogenic factors, such as pesticides, also impair immunity and can reduce chemical skin defences (146, 206). Compared to mammalian skin, frog skin has significantly greater uptake potential of xenobiotics that can bioconcentrate and may be detrimental to frog health (207–209). In some instances, the chemicals exert a direct effect on the skin epidermal cells. For example, short-term exposure of Italian pool frog (*Pelophylax bergeri*) skin cultures to cadmium resulted in alteration and disorganization of the skin epidermal layers, and ultimately induced cellular and molecular stress responses (210). In addition, exposure to environmental contaminants has been documented to directly affect the paracellular transport of ions across frog skin (211, 212), wherein cellular junctions play an important role in ion transport (2, 64, 75). Chemical contaminants can also impact host immune function resulting in altered host resistance to pathogens. Pesticide exposure has been shown to influence antiviral immunity in larval and adult frogs that led to increased susceptibility to pathogen invasion (213–215). It is then proposed that the potential for chemical contaminants to impact epidermal organization and alter frog skin permeability leads to increased pathogen susceptibility and host mortality. In general, while these studies are comprehensive at analysing either the impact of contaminants on amphibian skin or effect on ion permeability and pathogen susceptibility, none appear to directly report the regulation of cellular junctions in combination with pathogen susceptibility. Besides the effects of pesticides on skin permeability and pathogen susceptibility, specific pesticides such as carbaryl, have also been shown to significantly reduce frog skin peptide levels, but not bioactivity (146, 216).

### UV Radiation

Overexposure of frogs to UV-B radiation, in part due to deforestation and habitat loss, results in damage to the epidermal layer of larval and adult frogs (217, 218). Skin damage is characterized by epidermal shedding and sore formation, causing pronounced detrimental effects to maintenance of skin integrity and to physiological processes such as water and ion transportation (217, 218). Though largely unexplored in frogs, it is suggested that UV radiation breaches the skin barrier and induces host immunosuppression, causing the frog to be more susceptible to both pathogen invasion and exposure to chemical contaminants, leading to host mortality (218). Simultaneous exposure of larval *X. laevis* to pesticides and UV-B radiation resulted in higher mortality and instances of malformations, including those of the skin (208, 219). The interplay between UV and chemical exposure on frog skin immunocompetence, however, is not well-studied. While extensive research has been conducted in mammalian and fish models to elucidate the impact of irradiation on skin barrier integrity (220, 221), this is largely lacking in amphibian models.

### Pathogens

Much of our understanding of frog skin-pathogen interactions with FV3 and *Bd* derives from studies using *X. laevis* as a model (17). FV3 is transmitted through the environment, either through direct contact, indirect contact or consumption of infected carcasses (222, 223) and therefore must cross either the skin epithelial barrier or the gut epithelial barrier. Adult *X. laevis* are relatively resistant to FV3 and generally recover from mild symptoms 3–4 weeks after infection (18, 224, 225), whereas tadpoles are highly susceptible to FV3 infection (226). While the majority of *X. laevis*-FV3 research has bypassed the skin barrier via intraperitoneal injection of virus into the host (18, 227–229), water-bath exposure of healthy tadpole and adult *X. laevis* to FV3-infected frogs in the same tank revealed that healthy individuals become infected with FV3 within 3 h of exposure (230). A key symptom of FV3 infection in susceptible developmental stages or frog species is the formation of skin lesions, skin shedding, and epidermal cell necrosis (231, 232). It is proposed that loss of the skin barrier during FV3 infection allows for increased pathogen entry and ultimately leads to mortality in susceptible hosts, stressing the overall importance of the skin barrier and barrier integrity. While the precise contribution of frog skin innate immunity to FV3 resistance is unclear, initial studies suggest the initiation of a type I interferon response in the skin tissue of adults, compared to a type III interferon response in the skin of susceptible tadpoles, is important in conferring protection against FV3 viral entry and replication, and host mortality outcomes (181, 233).

Infection of susceptible frogs with *Bd* results in the disruption and cellular death of epidermal layers, resulting in host mortality (77, 234, 235). Comprehensive transcriptomic analyses on the skin of frogs infected with *Bd* revealed significant transcriptional regulation in the skin with generalized decreases in collagen, fibrinogen, elastin and keratin pathway transcript abundance, which corroborates with the observed disruption in epidermal skin integrity and loss of osmotic balance (236). Furthermore, a generalized lack of gene upregulation for key pro-inflammatory genes was observed, and instead an increase in transcripts for anti-inflammatory markers such as NF-κB inhibitors were seen (236) suggesting *Bd* may possess immunosuppressive capacity to limit frog skin innate immune defences and activation of underlying immune cells. Overall, these studies somewhat parallel observations in skin from FV3-infected frogs and suggests the loss of skin structural integrity may allow for increased pathogen entry and host mortality.

With the new era of transcriptomics approaches, untargeted transcriptomic molecular approaches have unveiled new insights into the impressive array of physiological functions performed by amphibian mucosal skin epithelium. Recent studies have analysed and compared the transcriptome of 3 anuran families to unveil genes involved in biosynthesis, metabolism, immunity, defence processes, and identification of antimicrobial peptides (123). In addition, transcriptomic studies have been performed on *Ranidae* and *Centrolenidae* frogs, species that are largely susceptible to pathogens plaguing amphibian populations, and included skin-specific immune gene expression analysis (125, 237, 238). However, the underlying molecular basis and mechanisms governing resistance and susceptibility of frog species are not well-understood. Further comparisons of frog skin transcriptomes from resistant and susceptible frogs will aid in elucidating the contribution of amphibian skin to resistance against lethal amphibian pathogens.

## Microbiome

In mammals, the skin microbiome plays a significant role in the defence against pathogens, injury and infection (239). Recently, attention has turned to elucidating the contribution of the frog skin microbiome in innate immune defences to emerging infectious diseases of amphibians, and in particular to *Bd*. The frog skin microbiome is seeded by microbes in the external environment (240–242) and shaped by the selective skin microenvironment (241, 243, 244). External contributors to the frog microbiome are the aquatic and soil environments (107) that are believed to serve as a reservoir for the frog skin microbiota (245–248), though horizontal transmission (e.g., during mating) (249), or vertical transmission (i.e., parent to offspring, although not common) (244) are also potential sources. In general, the main bacterial phyla found on frog skin consists mainly of Proteobacteria and Actinobacteria, however, this may vary across frog species, habitat and environmental factors (250–252). Not surprisingly, the frog skin microbiome is influenced by life stage (253), body region (254, 255), diet (254), capture site (256), habitat, captivity (254, 257), exposure to anthropogenic contaminants (258, 259), and treatment with antibiotics (260). While some of these factors may directly influence commensal skin microbes, it is possible that these same factors influence AMP gene expression, secretion of AMPs onto the skin, and AMP bioactivity. Initial studies have shown that the presence of commensal frog skin microbes is important for AMP synthesis (129). Thus, in light of the documented antimicrobial activity of many frog AMPs (Tables 1–4), the altered levels and activities of frog AMPs on the skin may also contribute to alteration of frog skin microbial communities. Depending on the conditions, skin microbiome dysbiosis may contribute to disease susceptibility in frogs, as observed in other vertebrates (252).

As in other vertebrates (239), symbiotic bacteria on frog skin appear to play an important role in defence against invading pathogens. Investigations of frog skin commensal microbes have revealed certain commensal bacteria to produce metabolites with anti-*Bd* activity (128, 241, 242, 261, 262). The frog skin commensal bacteria that produce anti-fungal metabolites are documented in the Antifungal Isolates Database (120, 263). Interestingly, metabolites produced by bacteria present on frog skin can also synergize with AMPs on the skin to inhibit *Bd* (264). Despite the exciting advances in the contribution of frog skin microbial communities to innate immune functions of frog skin, much remains to be elucidated in terms of host-microbiome-environment interplay.

## Concluding Remarks and Future Perspectives

Research on the innate immune functions of amphibian skin is emerging and beginning to shift from silos (e.g., investigating skin structure, AMPs or microbiomes) to integrative studies in which multiple facets of skin innate immunity are considered. This approach is critical to elucidating the complex host-pathogen-environment interactions at the skin interface that are participating in amphibian susceptibility to emerging infectious diseases and underpin the global decline in amphibian populations. However, it is evident from the literature that large knowledge gaps exist within each of the skin innate immune barrier silos and in understanding the intricate web of cellular and molecular mechanisms that function to maintain skin homeostasis and rapidly fend against pathogen insult and/or mediate wound healing. We believe there exists an imminent need to unravel the contribution of physical, chemical, cellular and microbiological barriers, to the innate immune function of amphibian skin and the abiotic and biotic environmental factors that regulate skin immunocompetency. Research on the presence and regulation of skin epithelial cell junction proteins under normal and stress conditions would provide vital information on which junction proteins are involved in skin epithelial cell junctions and under what conditions these junction proteins may be controlled to regulate skin permeability. The involvement of the diversity of junction proteins in amphibian skin barrier function is unknown. Little is known of the epithelial cells themselves in terms of the expressions of pattern recognition receptors, the localization of surface receptors (e.g., presence on apical or basal membrane), the role of epithelial cells in the direct sensing of non-self (and distinguishing commensal vs. pathogenic microbes) and in the initiation of innate immune responses leading to the direction of adaptive immune responses. Scrutiny of the literature yielded little information on amphibian PRRs themselves, save for their presence in the frog genome and apparent overall conservation of the signalling pathways as determined by molecular evolutionary analyses. The functional identification of PRR ligands, signalling pathways and downstream gene targets remains untouched. While the identification of amphibian AMPs and the characterization of their antimicrobial activity to human pathogens has been a topic of extensive investigation, comparatively little has been done to examine the antimicrobial activity of frog AMPs on frog pathogens. Virtually nothing is known of their contribution to amphibian skin wound healing or putative innate immune modulation functions, and if present, the receptors through which they bind, the signalling pathways they activate or the gene targets they regulate the expression of. An increasing number of researchers are surveying the commensal microbes present on frog skin, how frog skin microbial communities change with species, life stage, environment and presence of pathogens, yielding insight into the role of these microbes in defending against pathogenic insult. Yet much remains to be uncovered regarding how the frog host creates a permissive niche for certain microbial species while restricting others. Microbe-microbe interactions may also contribute to establishment of “healthy microbiomes” and deeper investigation into the metabolic capacities of commensal microbes will likely yield insight into the maintenance of certain microbial communities. Perhaps further characterization of the skin microbiome may foster the development of deployable “environmental probiotics” to habitats in which threatened or endangered amphibians reside as a way to seed the amphibian skin microbiome, thereby aiding in commensal microbe-mediated defence against frog pathogens. Achieving a complete understanding of skin innate immune function and the factors that affect skin barrier homeostasis may inform environmental policies aimed at conservation of amphibians to mitigate detrimental stressors that alter skin integrity and innate immune competency, or to develop strategies to safeguard threatened amphibians from further disease and population declines.

## Author Contributions

All authors listed have made a substantial, direct and intellectual contribution to the work, and approved it for publication.

### Conflict of Interest Statement

The authors declare that the research was conducted in the absence of any commercial or financial relationships that could be construed as a potential conflict of interest.

## Abbreviations

AMP: antimicrobial peptide
Bd: bactrachochytrium dendrobatidis
CCRs: chemokine receptors
CLRs: C-type-lectin like receptors
CPF: caerulein precursor fragment
DAMPs: damage-associated molecular patterns
dsRNA: double stranded RNA
EGFR: epidermal growth factor receptor
EK: Eberth-Katschenko
FPLR1: formyl peptide receptor-like-1
FV3: frog virus 3
GLP-1: glucagon-like peptide 1
GPCR: G-protein coupled receptor
hBD: human beta defensin
HDP: host defence peptide
IC50: inhibitory concentration 50
LGP2: laboratory of genetics and physiology 2
LPS: lipopolysaccharide
MAMPs: microbe-associated molecular patterns
MDA5: melanoma differentiation-associated gene 5
MIC: minimum inhibitory concentration
NF-κB: nuclear factor kappa-beta
NLR: nucleotide-binding oligomerization domain- (NOD-) like receptor
NOD: nucleotide-binding oligomerization domain
P2X7: purinergic receptors
PAMP: pathogen-associated molecular pattern
PGLa: peptide glycine-leucine-amide
PRR: pattern recognition receptor
RIG-I: retinoic acid inducible gene-I
RLR: RIG-I-like receptor
STAT3: signal transducer and activator of transcription 3
TLR: TOLL-like receptor
TLR: TOLL-like receptor
XPF: xenopsin precursor fragments.
